# Functionalization of Framboidal Phenylboronic Acid-Containing Nanoparticles via Aqueous Suzuki–Miyaura Coupling Reactions

**DOI:** 10.3390/molecules28083602

**Published:** 2023-04-20

**Authors:** André J. van der Vlies, Urara Hasegawa

**Affiliations:** Department of Materials Science and Engineering, Pennsylvania State University, 331 Steidle Building, University Park, State College, PA 16801, USA; amv5829@psu.edu

**Keywords:** phenylboronic acid, nanoparticles, aqueous Suzuki–Miyaura coupling, drug delivery

## Abstract

Polymeric nanoparticles with reactive functional groups are an attractive platform for drug carriers that can be conjugated with drugs through a cleavable covalent linkage. Since the required functional groups vary depending on the drug molecule, there is a need for development of a novel post-modification method to introduce different functional groups to polymeric nanoparticles. We recently reported phenylboronic acid (PBA)-containing nanoparticles (BNP) with a unique framboidal morphology created via one-step aqueous dispersion polymerization. Since BNPs have high surface area due to their framboidal morphology and contain a high density of PBA groups, these particles can be used as nanocarriers for drugs that can bind to PBA groups such as curcumin and a catechol-bearing carbon monoxide donor. To further explore the potential of BNPs, in this article we report a novel strategy to introduce different functional groups to BNPs via the palladium-catalyzed Suzuki–Miyaura cross-coupling reaction between the PBA groups and iodo- and bromo-coupling partners. We developed a new catalytic system that efficiently catalyzes Suzuki–Miyaura reactions in water without the need for an organic solvent, as confirmed by NMR. Using this catalyst system, we show that BNPs can be functionalized with carboxylic acids, aldehyde, and hydrazide groups while keeping their original framboidal morphology as confirmed via IR, alizarin red assay, and TEM. Furthermore, the potential of the functionalized BNP in drug delivery applications was demonstrated by conjugating the hydrogen sulfide (H_2_S)-releasing compound anethole dithiolone to carboxylic acid-functionalized BNPs and show their H_2_S-releasing capability in cell lysate.

## 1. Introduction

Polymeric nanoparticles have been extensively investigated as drug carriers for overcoming biological barriers to achieve efficient and safe drug delivery. Those particles can be loaded with therapeutic agents via physical encapsulation or chemical conjugation. Despite the simplicity of the physical encapsulation method, this drug-loading strategy often results in premature leakage of drugs before reaching the target tissue. To solve this issue, chemical conjugation of drugs to polymeric nanocarriers via a biodegradable linker has emerged as an alternative method that can reduce drug leakage during blood circulation. With this approach, targeted drug release can be made possible by choosing a linker that can be cleaved in response to biological triggers such as pH change, oxidative stress, and reductions in the microenvironment. However, since the functional groups for conjugation depend on the chemical structure of the drug, it is critical to design nanoparticles containing reactive groups that allow for conjugation of a particular drug. Therefore, a polymeric nanoparticle platform allowing for modular post-modification is of great interest in the drug delivery field.

We previously reported synthesis of phenylboronic acid (PBA) containing nanoparticles with framboidal morphology (BNP) via aqueous dispersion polymerization of 3-(acrylamido)phenylboronic acid (APBA) in phosphate buffer solution using methoxy-poly(ethylene glycol) acrylamide (mPEG-AM) as a polymerizable steric stabilizer, methylenebisacrylamide (MBA) as a crosslinker, and ammonium persulfate (APS) as an initiator [1]. We found that monodisperse clusters composed of small primary particles of ca. 20 nm (framboidal particle) were formed during the polymerization reaction. Due to the presence of PBA groups with a pKa of 8.5 [2], the BNPs show a reversible swelling/deswelling behavior in response to changes in pH. The BNPs are amendable to surface modification by replacing mPEG-AM with a carboxylic acid-terminated PEG-AM. The carboxyl groups on the BNP’s surface were modified with mannosamine via aqueous carbodiimide coupling chemistry. These mannosylated BNPs showed increased cellular uptake in macrophages via the mannose receptor [3]. In addition, the BNPs could be loaded with a catechol-bearing carbon monoxide (CO) donor through boronate ester formation between the PBA groups of the BNPs and catechol [4]. A high loading capacity of water-soluble CO-donors could be achieved due to the higher relative surface area of the framboidal morphology, which increases the amount of PBA moieties exposed to the aqueous media. We showed that these CO-donor-loaded BNPs suppressed the inflammatory response in macrophages. Furthermore, BNPs showed strong binding capability with curcumin, a plant-derived antioxidant with a wide variety of bioactivities [5]. The BNP mitigated the well-known degradation of this drug in aqueous solutions. The curcumin-loaded BNPs showed antiangiogenic and anticancer effects in the CAM assay.

**Figure 1:**
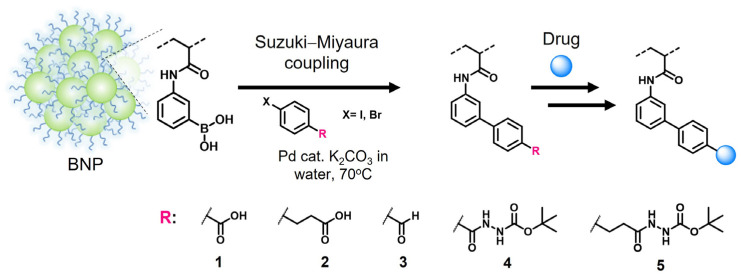
Functionalization of phenylboronic acid-containing framboidal nanoparticles via aqueous Suzuki–Miyaura coupling reaction with aryl halides containing different functional groups: (**1** and **2**) carboxylic acids, (**3**) aldehyde, (**4** and **5**) Boc-protected hydrazides.

While BNPs show great promise as drug carriers, their applications are limited to drugs that are capable of binding to PBA reversibly. To circumvent this limitation, we sought to convert the PBA groups of BNPs to different functional groups that would allow for covalent attachment of drug molecules. Apart from the interaction with diols, the PBA groups are widely used as chemical building blocks in organic synthesis. It is known that the PBA groups react with aryl halides in the presence of a Pd catalyst to form C–C bonds via Suzuki−Miyaura cross-coupling reactions. We hypothesize that this reaction allows for the introduction of various reactive groups to BNPs as outlined in Figure 1. Here we present the functionalization of BNPs via aqueous Suzuki–Miyaura cross coupling reactions using a new water-soluble palladium/phosphine catalytic system. The BNPs were reacted with a series of aryl halides and characterized via an alizarin red (AZR) fluorescence assay, dynamic light scattering (DLS), transmission electron microscopy (TEM), UV-Vis spectroscopy (UV-Vis), and infrared spectroscopy (IR). Furthermore, BNPs bearing carboxyl groups were modified with an anethole dithiolethione (ADT) derivative and a gasotransmitter hydrogen sulfide (H_2_S) donating drug, following which their H_2_S release capability was tested.

## 2. Results

### 2.1. Aqueous Suzuki–Miyaura Coupling Model Reactions

The Suzuki–Miyaura cross-coupling reaction is the cross-coupling of boronic acids with organo-halides catalyzed via a palladium (Pd(0))/ligand complex. [6] Typically, this reaction is carried out in an organic solvent or organic solvent/water mixture, with the organic solvent making up the larger portion as measured by volume. In organic solvent/water mixtures, a palladium/phosphine catalyst system, such as Pd(PPh_3_)_4_, is commonly used. However, the organic solvent as well as the water-insoluble catalyst must be removed from functionalized nanomaterials in order to use them in a biological setting. Therefore, it is of importance to develop a catalytic system for the Suzuki–Miyaura coupling reaction that can be carried out in 100% water. Thus far, only a few papers have reported Suzuki–Miyaura coupling reactions run in water only [7]. Due to the limited aqueous solubility of the catalyst, a majority of the reports used surfactants [8], reverse phase transfer catalyst [9], host-guest interactions [10] and hydrophilic ligands [11], palladium nanoparticles [12,13], supramolecular systems [14], or micelles [15]. However, those colloidal catalytic systems cannot be applied for nanoparticle modifications due to the difficulty in separating these catalyst systems from the functionalized nanoparticles, as well as their low reaction efficiency with the sterically hindered PBA groups on the nanoparticles. Therefore, a water-soluble palladium catalyst with hydrophilic ligands would be the best choice for avoiding these problems. Wallow et al. reported the synthesis of water-soluble Pd catalyst containing a sulfonated triphenylphosphine ligand, sodium diphenylphosphinobenzene-3-sulfonate PPh_2_PhSO_3_Na (Pd(PPh_2_PhSO_3_Na)_4_), from PdCl_2_ and hydrazine [16]. Inspired by this report, we hypothesized that simply mixing an aqueous solution of Na_2_PdCl_4_ and PPh_2_PhSO_3_Na followed by reduction of Pd^II^ to Pd^O^ with formic acid (HCOOH) would also result in an active catalyst.

**Scheme 1:**
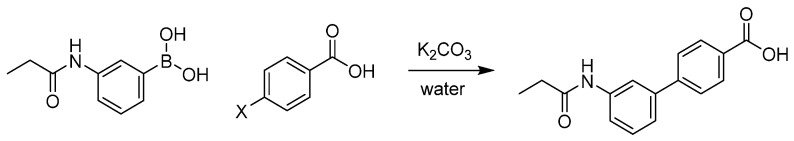
Model reactions to study the effect of reaction parameters on the Suzuki–Miyaura coupling reaction between the phenylboronic acid model compound and 4-bromo-benzoic acid (X = Br) or 4-iodo-benzoic acid (X = I) in water. Palladium catalyst system: Na_2_PdCl_4_/PPh_2_PhSO_3_Na/HCOOH, molar ratio 1:4:5.

To test our hypothesis, we carried out model reactions, as shown in Scheme 1. We used (3-propionamidophenyl)boronic acid, which resembles the chemical structure of the phenyl boronic acid groups present on the BNP, as the boronic acid model compound. The 4-iodo- or 4-bromo-benzoic acid and the boronic acid were dissolved in water and a solution of Na_2_PdCl_4_ and PPh_2_PhSO_3_Na was added (molar ratio 1:4), followed by formic acid (HCOOH) in five times molar excess to the amount of palladium. The reaction was run under argon in degassed water to avoid oxidation of the phosphine ligand. Conversion (%) was determined via ^1^H NMR after acidification with sodium hydrogen sulfate and lyophilizing the sample. Yields in % are those after working up the reaction mixture. The chemical identity of the product was confirmed via NMR and IR (see Supplementary Materials). 

As can be seen in Table 1, both the 4-iodo- and 4-bromo-benzoic acids (entries 3 and 10) led to 100% conversion of the boronic acid at 70 °C with 0.01% Pd relative to the amount of benzoic acid. Even at room temperature, conversion of 4-iodo-benzoic acid was quantitative with 0.1 % Pd (entry 5). The decreased reactivity of 4-bromo-benzoic acid compared to 4-iodo-benzoic acid is illustrated for 1% Pd at room temperature (entries 4 and 7). These results clearly show that the Na_2_PdCl_4_/PPh_2_PhSO_3_Na/HCOOH catalyst system is capable of catalyzing aqueous Suzuki–Miyaura coupling reactions.

### 2.2. BNP Modification via Aqueous Suzuki–Miyaura Coupling Reaction

**Figure 2:**
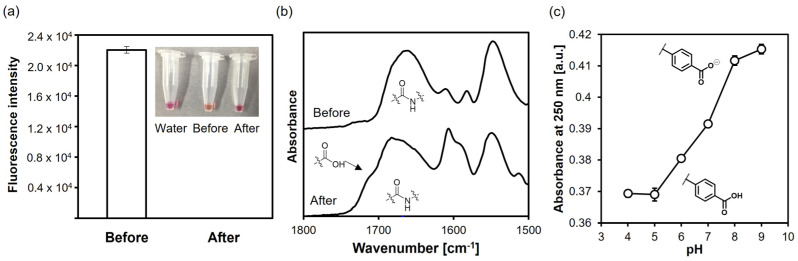
Modification of BNPs with 4-iodo-benzoic acid via aqueous Suzuki–Miyaura coupling reaction (NP-1). (**a**) PBA groups before and after the reaction as detected by the alizarin red S assay. The amount of the PBA group was determined by the formation of fluorescent PBA–alizarin red S complex. The samples were mixed with alizarin red S and the fluorescence intensity (*λ*_ex_ = 485 nm, *λ*_em_ = 616 nm) was measured. Insert: Photos of alizarin red S solutions. Left: without BNP. Middle: with BNP before reaction. Right: with BNP after reaction. (**b**) IR spectra of lyophilized BNPs before and after the Suzuki–Miyaura reaction. (**c**) pH-dependent change in absorbance at 250 nm of NP-1. *n* = 3.

Having shown successful model reactions, we next used the Na_2_PdCl_4_/PPh_2_PhSO_3_Na/HCOOH catalyst system for functionalizing BNPs with 4-iodobenzoic acid (NP-1). To assess successful functionalization of BNPs, we used the fluorescent alizarin red assay for quantifying the remaining PBA groups. This assay is based on the interaction of alizarin red with PBA that results in a fluorescent product. As shown in Figure 2a, the fluorescence was not observed after the reaction, showing full conversion of the PBA groups. Compared to the model reactions in Table 1, a catalyst loading of 1% Pd gave complete functionalization of the BNP. This higher loading is needed because of the lower PBA concentration in the BNP solution compared to the PBA concentration used for the model reactions, which results in a slower reaction rate. 

To show the presence of the coupling product of PBA groups with 4-iodo-benzoic acid, the BNP solution before and after the reaction was lyophilized and analyzed via infrared (IR) spectroscopy (Figure 2b). The IR spectrum of the BNPs before the reaction shows a broad absorption of the amide (1695–1630 cm^−1^) that corresponds to the amide vibrations of polymerized APBA, mPEG-AM, and MBA (Figure 2b). On the other hand, the BNPs after Suzuki–Miyaura coupling show a much broader signal with a shoulder at higher wavenumbers due to ν(C=O) vibrations of the carboxylic acid (Figure 2b). A similar spectral change was also observed for the product of the model reaction (Scheme 1) showing the overlapping amide and carboxylic acid ν(C=O) stretching vibrations at 1666 and 1672 cm^−1^ (Figure S1). Compared to the model reaction product, the stretching vibrations were observed at higher wavenumbers for BNPs, suggesting that the carboxylate groups have interactions with other functional groups present on the NP.

To further confirm the presence of the carboxyl groups, UV–Vis spectra of the functionalized BNP solutions were measured at different pH levels. Since the carboxylic acid is directly bound to the phenyl ring which shows a strong absorbance at 250 nm, the absorbance at this wavelength is expected to change with a change in pH. Indeed, UV–Vis measurements show a pH-dependent absorbance at 250 nm (Figure 2c). 

The diameter of the BNPs before and after the reaction was measured via DLS. As can be seen in Table 2 (Entry: NP-1), the Z-average diameter of the resulting NP increased from 101.5 to 130.5 nm, probably due to swelling of the functionalized BNPs by electrostatic repulsion of the negatively charged carboxylic acid groups. 

To further test the scope of BNP functionalization, we also used 3-(4-bromophenyl)propionic acid as the coupling partner (Table 2, NP-2). With this coupling partner, functionalization was near quantitative, but it required a higher palladium loading (5%) compared with 4-iodo-benzoic acid to achieve this degree of functionalization. This successful functionalization was also confirmed via IR (Figure S3). The NP-2 shows a shoulder at 1728 cm^−1^, well-separated from the broad amide band, that can be assigned to the carboxylate group. A similar spectral change was observed for the product of the model reaction (see Supplementary Materials, model compound (**2**)) showing the amide and carboxylic acid as two well-separated peaks at 1666 and 1689 cm^−1^, respectively (Figure S2). Furthermore, as observed for NP-1, the size increases from 101.7 to 119.2 nm, which we attribute to electrostatic repulsion of the carboxylate groups.

To demonstrate the modularity of our approach, we next used 4-iodo-benzaldehyde as the coupling partner. In addition to carboxyl groups, we used the Suzuki–Miyaura coupling partners with aldehyde and hydrazides, which allow for bioconjugate reactions to link drug and protein molecules to the NPs through different chemistries. Aldehyde groups can be used to conjugate drugs with hydroxylamines (oxime formation), hydrazides (hydrazone formation), and amines (Schiff base formation). Hydrazide groups can be used to conjugate aldehyde and ketone-containing drugs. 

As can be seen from Table 2 (NP-3), this compound was as reactive as 4-iodo-bezoic acid and resulted in a quantitative reaction with a Pd catalyst loading of 1%. The presence of the coupling product was confirmed via IR spectra (Figures S4 and S5). The product of the model reaction using 4-iodo-benzaldehyde showed two well-separated absorptions at 1693 and 1664 cm^−1^ that can be assigned to the ν(C=O) stretching vibrations of the aldehyde and the amide groups, respectively. The lyophilized NP-3 shows a shoulder at 1692 cm^−1^ in addition to the broad amide bands, suggesting successful conjugation. 

We also prepared BNPs functionalized with two Suzuki–Miyaura coupling partners containing a protected hydrazine group, as shown in Table 2 (NP-4 and NP-5). It was necessary to protect the hydrazine group with the tert-butoxy carbonyl (Boc) group because the use of free hydrazine resulted in no functionalization of BNPs according to the alizarin red assay, presumably due to catalyst inactivation. With these coupling partners, functionalization of BNPs occurred, albeit with lower degrees of functionalization (70–80%) and the requirement of palladium loadings of 5%. The presence of the Boc-protected hydrazine groups was inferred from the IR spectra (Figures S6–S9) showing broad absorptions at 1728 and 1732 cm^−1^, respectively, due to the ν(C=O) stretching vibration of the (CH_3_)_3_C(O)–NH group. Furthermore, both NP-4 and NP-5 were deprotected by cleaving the Boc group in 1 M HCl (aq). 

### 2.3. Framboidal Structures of Suzuki–Miyaura Coupling-Functionalized BNP

**Figure 3:**
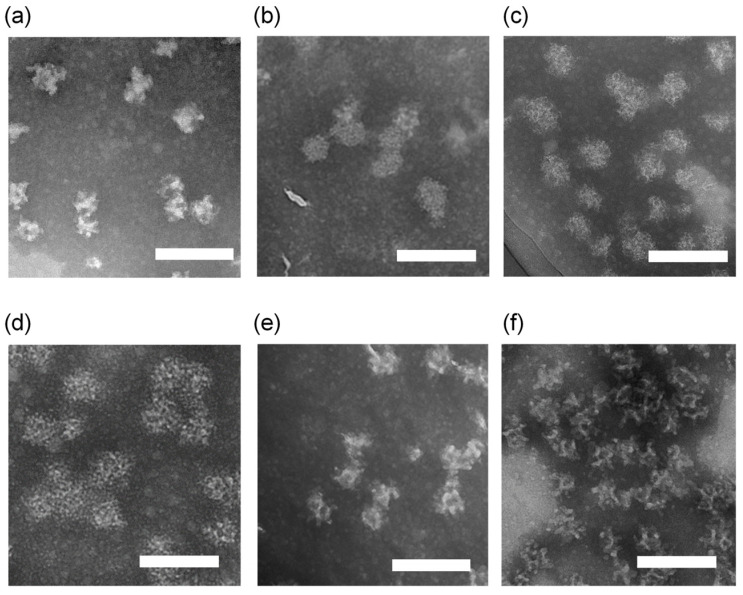
TEM images of BNP before and after the Suzuki–Miyaura cross-coupling reaction. (**a**) BNP (no modification), (**b**) NP-1, (**c**) NP-2, (**d**) NP-3, (**e**) NP- 4, and (**f**) NP-5. For NP-4 and NP-5, the Boc-protecting group was removed with HCl (aq) before acquiring the TEM images. Samples were negatively stained with 2 wt% sodium dodecatungstato(VI) phosphate solution. Scale bars: 200 nm.

Since we showed successful functionalization of BNPs upon Suzuki–Miyaura cross-coupling reactions, we were interested to see whether BNPs maintained their framboidal morphology after reaction. The aqueous solutions of BNPs before and after reaction were stained negatively and observed via TEM. As shown in Figure 3, all BNPs functionalized with the different coupling partners (NP-1 to NP-5) maintained their unique framboidal morphology.

### 2.4. Functionalization of Carboxylate NP (NP-1) with ADT

**Figure 4:**
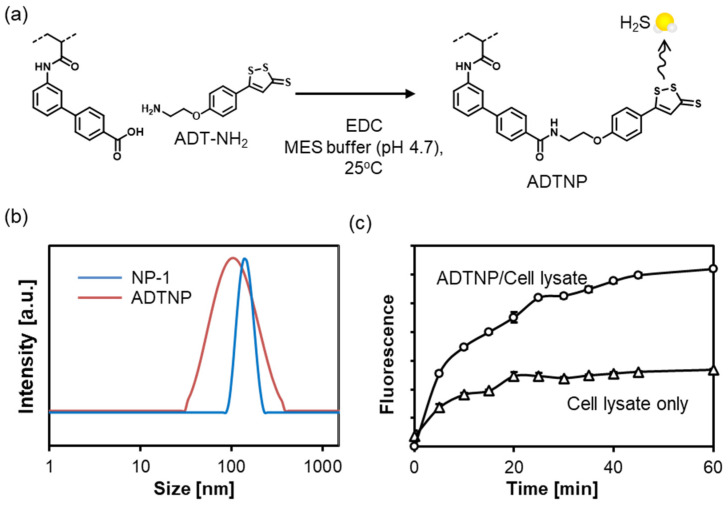
H_2_S donor ADT-conjugated nanoparticles (ADTNP). (**a**) Conjugation of a H_2_S donor ADT-NH_2_ with NP-1 via EDC coupling. (**b**) Size distribution before (NP-1) and after conjugation (ADTNP). (**c**) H_2_S release from ADTNP in cell lysate as detected by the WSP-1 H_2_S detection dye.

To show the potential of the functionalized BNPs in drug delivery applications, we conjugated ADT-NH_2_, an amine derivative of the widely used H_2_S donor molecule [17], to NP-1 (ADTNP) (Figure 4a). We functionalized NP-1 with ADT using the carbodiimide chemistry. Successful conjugation was confirmed by the strong absorbance of conjugated ADT at 436 nm, which is typical of the dithiolone structure. Functionalization of NP-1 with ADT-NH_2_ led to a decrease in size from 116.8 to 94.8 nm according to DLS (Figure 4b). This size decrease can be attributed to deswelling of the particles due to the presence of highly hydrophobic ADT. To show that ADTNPs were capable of releasing H_2_S, we incubated the ADTNP with cell lysate from mouse macrophages to induce H_2_S release from the ADT groups and monitored formation of H_2_S using the H_2_S-specific WSP-1 fluorescent dye [18]. As shown in Figure 4c, sustained H_2_S release was observed from ADTNPs over 1 h.

## 3. Materials and Methods

### 3.1. Instrumentation

NMR spectroscopy: ^1^H NMR spectra were measured with a Bruker DPX400 NMR spectrometer. A total of 32 scans were collected and the d1 was set to 1 s. The chemical shifts are reported relative to the residual undeuterated solvent signals at 7.26 (CDCl_3_) and 2.50 (*d_6_*-DMSO) ppm.

Dynamic light scattering (DLS): Hydrodynamic diameters of the micelles were obtained via an Otsuka instrument and disposable micro cuvettes. The Z-average diameter and polydispersity index (PDI) were calculated using the cumulant method.

Transmission electron microscopy (TEM): High-resolution carbon-coated cupper grids (STEM 100 grid) were purchased from Oken shouji. Sample solutions in water (5 μL) were placed onto carbon-coated 250 mesh copper grids and maintained for 5 min at RT. The grid was dried by blotting the side of the grid with a filter paper. The grids were negatively stained with 2 wt% sodium dodecatungstato(VI)-phosphate solution (5 μL) for 1 min. The grid was dried by blotting the side of the grid with a filter paper. Images were acquired with a Hitachi H-7700 microscope system operating at 100 kV. 

Infrared spectroscopy (FT-IR): Spectra were obtained with a Thermo Scientific Nicolet iS5 equipped with an iF5 universal ATR sample accessory. A total of 32 scans were collected at a resolution of 4 cm^−1^.

UV–Vis spectroscopy: Spectra were obtained with a Thermo Scientific Nanodrop One^c^ instrument or a Tecan infinite M200 plate reader using transparent 96-well polystyrene plates.

UV–Vis/fluorescence spectroscopy: Fluorescence intensities were measured with a Tecan infinite M200 plate reader using black 96-well polystyrene plates.

### 3.2. Suzuki–Miyaura Coupling Model Reactions with 4-Iodo- and 4-Bromo-Benzoic Acid

The Suzuki–Miyaura coupling partners, 4-iodo-benzoic acid (1) or 4-bromo-benzoic acid (2) (0.25 mmol, 1 eq) and (3-propionamidophenyl)boronic acid (0.26 mmol, 1.05 eq) and K_2_CO_3_ (69.8 mg, 0.50 mmol, 2.0 eq), were dissolved in 10 mL water that had been degassed by bubbling nitrogen for at least 15 min. The Schlenk tube was closed with a rubber septum and the system evacuated and purged with argon three times in total. Under a positive argon gas pressure, a solution of Na_2_PdCl_4_ and PPh_2_PhSO_3_Na (100 μL) in water and a solution of HCOOH (100 μL) in water were added. The Na_2_PdCl_4_/PPh_2_PhSO_3_Na (molar ratio 1:4) solution was made by mixing a freshly prepared solution of PPh_2_PhSO_3_Na in degassed water with a solution made by diluting a 100 mM stock solution of Na_2_PdCl_4_ (stored at −20 °C) with water. The HCOOH solution (5 eq relative to Pd) was prepared freshly each time. The Schlenk tube was then closed with a rubber septum and stirred for 24 h at the indicated temperature. Part of the reaction mixture (1 mL) was acidified with 0.1 mL 1 M NaHSO_4_ (aq) and lyophilized. The resulting solid was suspended in 700 μL *d_6_*-DMSO, filtered through a plug of glass wool, and measured via ^1^H NMR to determine the % conversion (Table 1). The remainder of the reaction mixture (9 mL) was acidified with 1 M NaHSO_4_ (aq) (1 mL) and the suspension filtered through a glass filter. After washing with H_2_O (3 × 30 mL), the solid was dissolved in acetone (30 mL), and, after solvent removal, dried under vacuum.

### 3.3. Synthesis of BNPs

BNPs were synthesized as reported previously [1]. mPEG-AM (67.9 mg, 5kDa), APBA (9.5 mg), and MBA (0.9 mg) were dissolved in 18.75 mL phosphate buffer (pH 7) and evacuated and purged with argon (3×). The solution was then heated at 70 °C for 1 h before adding 60 μL degassed water containing APS (1.15 mg) under argon flow. After 20 h, the blueish solution was transferred to dialysis tubing (MWCO 3.5 kDa) and dialyzed against water (5 L) for 1 d with regular replacement of the water. 

### 3.4. Alizarin Red Fluorescence Assay

To confirm the degree of functionalization of the BNP after Suzuki–Miyaura coupling reactions, i.e., the absence of PBA groups after reaction, 10 μL of the reaction mixture before and after reaction was diluted with 10 μL alizarin red solution in water (1.1 mM) and 180 μL phosphate buffer with a pH of 8. After 2 h, the fluorescence was measured using λ_excitation_ = 485 nm and λ_emission_ = 616 nm. After subtracting the background due to the buffer alone, the fluorescence intensity after reaction was divided by the fluorescence intensity of the BNP and expressed as a % conversion. *n* = 3. 

### 3.5. BNP Functionalization with 4-Iodo-Benzoic Acid (NP-1)

BNP (10 mL, PBA = 1.6 mM, 0.016 mmol PBA groups, 1 eq) was added to 4-iodo-benzoic acid (19.4 mg, 0.078 mmol, 4.9 eq) and K_2_CO_3_ (21 mg, 0.15 mmol, 9.9 eq), and the solution was degassed for 15 min via bubbling argon. The Schlenk tube was closed with a septum and evacuated and purged with argon three times. Under argon flow, an aqueous solution (100 μL) containing Na_2_PdCl_4_/PPh_2_PhSO_3_Na (0.01 eq Pd) and an aqueous solution (100 μL) containing HCOOH (0.05 eq) were added. The mixture was again evacuated and purged with argon 3× and then stirred at 70 °C for 32 h. After cooling down, the alizarin red assay showed 100% conversion of the PBA groups. The mixture was transferred to dialysis tubing with MWCO 3400 kDa and dialyzed for 3 days against 4 L water with regular replacement of the water. DLS (water) before Suzuki–Miyaura: Z-avg = 101.5 nm, PDI = 0.028); after Suzuki–Miyaura: Z-avg = 130.5 nm, PDI = 0.116.

### 3.6. BNP Functionalization with 3-(4-Bromophenyl)propionic Acid (NP-2)

BNP (5 mL, PBA = 1.9 mM, 0.0095 mmol PBA groups, 1 eq) was added to 3-(4-bromophenyl)propionic acid (11 mg, 0.046 mmol, 4.9 eq), and to the suspension was added an aqueous solution (100 uL) of K_2_CO_3_ (13 mg, 0.093 mmol, 9.7 eq). The clear solution was degassed for 15 min via bubbling argon. The Schlenk tube was closed with a septum and evacuated and purged with argon three times. Under argon flow, an aqueous solution (100 μL) containing Na_2_PdCl_4_/PPh_2_PhSO_3_Na (0.05 eq Pd) and an aqueous solution (100 μL) containing HCOOH (0.25 eq) were added. The mixture was again evacuated and purged with argon 3× and then stirred at 70 °C for 42 h. After cooling down, the alizarin red assay showed 97% conversion of the PBA groups. The mixture was transferred to dialysis tubing with MWCO 3400 kDa and dialyzed for 3 days against 4 L water with regular replacement of the water. DLS (water) before Suzuki–Miyaura coupling: Z-avg = 101.7 nm, PDI = 0.058); after Suzuki–Miyaura coupling: Z-avg = 119.2 nm, PDI = 0.091.

### 3.7. BNP Functionalization with 4-Iodobenzaldehyde (NP-3)

BNP (5 mL, PBA = 3.2 mM, 0.016 mmol PBA groups, 1 eq) was added to 4-iodobenzaldehyde (19 mg, 0.083 mmol, 5.1 eq) and K_2_CO_3_ (12 mg, 0.090 mmol, 5.6 eq). The suspension was carefully degassed for 15 min via bubbling argon. The Schlenk tube was closed with a septum and evacuated and purged with argon three times. Under argon flow, an aqueous solution (100 μL) containing Na_2_PdCl_4_/PPh_2_PhSO_3_Na (0.01 eq Pd) and an aqueous solution (100 μL) containing HCOOH (0.05 eq) were added. The mixture was again evacuated and purged with argon 3× and then stirred at 70 °C for 43 h. After cooling down, the alizarin red assay showed 100% conversion of the PBA groups. The mixture was transferred to dialysis tubing with MWCO 3400 kDa and dialyzed for 3 days against 4 L water with regular replacement of the water. DLS (water) before Suzuki–Miyaura coupling: Z-avg = 237.5 nm, PDI = 0.077); after Suzuki–Miyaura coupling: Z-avg = 234.6 nm, PDI = 0.032.

### 3.8. BNP Functionalization with Suzuki–Miyaura Coupling Partner ***4*** (NP-4) 

BNP (5 mL, [PBA] = 1.9 mM, 0.0092 mmol PBA groups, 1 eq) was mixed with Suzuki–Miyaura coupling partner (**4**) (see Supplementary Materials for its synthesis) (17 mg, 0.047 mmol, 5 eq), and to the suspension was added an aqueous solution (100 uL) of K_2_CO_3_ (6.4 mg, 0.046 mmol, 5 eq). The suspension was carefully degassed for 15 min via bubbling argon. The Schlenk tube was closed with a septum and evacuated and purged with argon three times. Under argon flow, an aqueous solution (100 μL) containing Na_2_PdCl_4_/PPh_2_PhSO_3_Na (0.05 eq Pd) and an aqueous solution (100 μL) containing HCOOH (0.25 eq) were added. After another evacuation and purge with argon 3×, the clear solution was placed at 70 °C and reacted for 42 h. After cooling to room temperature, the mixture was filtered through glass wool to remove unreacted Suzuki–Miyaura coupling partner (**4**). After cooling down, the alizarin red assay showed 79% conversion of the PBA groups, and the filtrate was dialyzed against water (1 L) and MWCO 3500 for 3 days. DLS (water) before Suzuki–Miyaura coupling: Z-avg = 101.7 nm, PDI = 0.058; after Suzuki–Miyaura coupling: Z-avg = 113.4 nm, PDI = 0.214.

### 3.9. BNP Functionalization with Suzuki–Miyaura Coupling Partner ***5*** (NP-5)

BNP (5 mL, [PBA] = 1.89 mM, 0.0092 mmol PBA groups, 1 eq) was mixed with Suzuki–Miyaura coupling partner (**5**) (see Supplementary Materials for its synthesis) (16 mg, 0.046 mmol, 5 eq), and to the suspension was added an aqueous solution (100 uL) of K_2_CO_3_ (6.4 mg, 0.046 mmol, 5 eq). The suspension was carefully degassed for 15 min via bubbling argon. The Schlenk tube was closed with a septum and evacuated and purged with argon three times. Under argon flow, an aqueous solution (100 μL) containing Na_2_PdCl_4_/PPh_2_PhSO_3_Na (0.05 eq Pd) and an aqueous solution (100 μL) containing HCOOH (0.25 eq) were added. After another evacuation and purge with argon 3x, the clear solution was placed at 70 °C and reacted for 42 h. After cooling to room temperature, the mixture was filtered through glass wool to remove unreacted Suzuki–Miyaura coupling partner (**5**). After cooling down, the alizarin red assay showed 72% conversion of the PBA groups and the filtrate was dialyzed against water (1 L) and MWCO 3500 for 3 days. DLS (water) before Suzuki–Miyaura coupling: Z-avg = 101.7 nm, PDI = 0.058); after Suzuki–Miyaura coupling: Z-avg = 102.4 nm, PDI = 0.080.

### 3.10. Modification of NP-1 with ADT-NH_2_ (ADTNP)

NP-1 after dialysis was used for this experiment. Based on the original volume (and PBA concentration) and the final volume after Suzuki–Miyaura coupling and dialysis (determined gravimetrically using a density of 1 g/mL), the COOH concentration was estimated under the assumption that no material was lost during the dialysis step. NP-1 (2 mL, [COOH] = 4 mM, 0.008 mmol PBA groups, 1 eq) was mixed with MES (48.2 mg), and the pH was adjusted with NaOH (aq) to a pH of 4.7 (final MES concentration = 112 mM). To the solution was added solid ADT-NH_2_ TFA salt (4 mg, 0.010 mmol, 1.2 eq), and the solution was homogenized using a pipette. Then, EDC (49.2 mg, 0.26 mmol, 32 eq) was added, and the turbid solution became clear. After reacting for 3 h, additional EDC (75.8 mg, 0.40 mmol, 50 eq) was added. After stirring for another 2 h, the reaction mixture was transferred to a dialysis tube (MWCO 3.5kDa) and dialyzed until the water remained colorless due to the absence of free ADT-NH_2_. DLS after ADT-conjugation: Z-avg = 94.8 nm, PDI = 0.215).

### 3.11. Quantification of ADT

Nanoparticle solutions were diluted with DMF (1:9) and the absorbance at 436 nm was measured via UV–Vis. The concentration was determined by comparing against a standard of ADT in the same solvent mixture.

### 3.12. Cell Lysate Preparation

Cell lysate was prepared as previously reported [18]. RAW-Blue macrophages (80% confluent) were scraped off from the culture flask and centrifuged at 500 rpm for 5 min. The cell pellet was washed with cold Dulbecco’s phosphate-buffered saline (PBS) three times. The cell suspension was centrifuged at 500 rpm for 5 min, resuspended in passive lysis buffer at 3.8 × 10^6^ cells/mL, and vortexed at room temperature for 10 min. The suspension was centrifuged, and the clear supernatant was collected and stored at −20 °C. 

### 3.13. Measurement of H_2_S Release in Cell Lysate via WSP-1 Fluorescent H_2_S Detection Dye

H_2_S release from the ADTNP was measured as reported previously [18]. To a black well polystyrene plate containing 100 μL of 20 vol% cell lysate/PBS pH 7.4 was added 10 μL of ADTNP and 10 μL of WSP-1 in acetonitrile (final concentration: 100 μM). As a control, cell lysate without the donor (10 μL water) was measured as well. Immediately after adding, the fluorescence was measured with a Tecan well plate reader (e_xcitation_ = 465 nm, λ_emission_ = 515 nm).

## 4. Conclusions

BNPs were functionalized via the Suzuki–Miyaura coupling reaction between the PBA groups of BNPs and aryl halides in water without the need for an organic solvent. Key was the use of a new catalytic system that consists of readily available reagents sodium tetrachloropalladate (Na_2_PdCl_4_) and the water-soluble phosphine ligand sodium diphenylphosphinobenzene-3-sulfonate (PPh_2_PhSO_3_Na), which can be mixed with BNPs in water followed by the addition of formic acid (HCOOH) in excess over Na_2_PdCl_4_ to promote reduction of Pd^II^ to Pd^0^. This catalyst system efficiently leads to cross-coupling of the BNP with a series of coupling partners which have carboxylic acid, aldehyde, and Boc-protected hydrazide groups. Importantly, the unique framboidal morphology of the BNPs was maintained after the Suzuki–Miyaura coupling reaction. Furthermore, BNPs functionalized with carboxyl groups can be further conjugated with amine-containing molecules, such as the H_2_S donor ADT, using aqueous carbodiimide chemistry. This post-modification approach can be used to conjugate different drug molecules, not limited to small drug molecules, but also proteins and nucleic acids.

## Figures and Tables

**Table 1:** Effect of catalyst loading, reaction temperature, and coupling partner on the conversion of the model reaction shown in Scheme 1.

Entry	Pd/B [mol %] ^1^	T [°C] ^2^	X	Conversion [%] ^3^	Yield [%] ^4^
1	1	70	I	100	94
2	0.1	70	I	100	79
3	0.01	70	I	100	93
4	1	25	I	100	98
5	0.1	25	I	100	91
6	0.01	25	I	47	n.d.
7	1	25	Br	78	n.d.
8	1	70	Br	100	98
9	0.1	70	Br	100	99
10	0.01	70	Br	100	100

^1^ Palladium catalyst system: Na_2_PdCl_4_/PPh_2_PhSO_3_Na/HCOOH, molar ratio 1:4:5. ^2^ Reactions were run in water in the presence of K_2_CO_3_ under argon for 24 h at the indicated temperature. ^3^ Part of the reaction mixture was acidified with NaHSO_4_ (aq), lyophilized, and measured via ^1^H NMR to determine the % conversion (see experimental section for details). ^4^ Yields were determined gravimetrically after working up the reaction (see experimental section for details).

**Table 2:** Modification of PBANP via aqueous Suzuki–Miyaura coupling reaction.

					DLS before Suzuki		DLS after Suzuki	
Entry	Coupling Partner	Pd/B [%] ^1^	Time [h]	Conversion [%] ^2^	Diameter [nm] ^3^	PDI ^3^	Diameter [nm] ^3^	PDI ^3^
NP-1	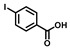	1	32	100	101.5	0.028	130.5	0.116
NP-2	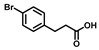	5	42	97	101.7	0.058	119.2	0.091
NP-3	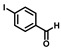	1	43	100	237.5	0.077	234.6	0.032
NP-4	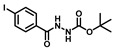	5	42	79	101.7	0.058	113.4	0.214
NP-5	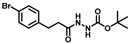	5	42	72	101.7	0.058	102.4	0.080

^1^ The molar ratio of Na_2_PdCl_4_ to PBA in the reaction mixture; PPh_2_PhSO_3_Na to Na_2_PdCl_4_ molar ratio = 4, HCOOH to Na_2_PdCl_4_ molar ratio = 5, coupling partner to PBA molar ratio = 5, K_2_CO_3_ to PBA molar ratio = 10 (entries 1–3) or 5 (entries 4–5), reaction temperature 70 °C. ^2^ Measured via fluorescence with the alizarin red assay. ^3^ Determined via DLS. Z-average diameter and polydispersity index (PDI) was calculated using the cumulant method.

## Data Availability

Not applicable.

## Supplementary Materials

The following supporting information can be downloaded at: https://www.mdpi.com/article/10.3390/molecules28083602/s1, Synthesis, IR and 1H NMR spectra of the Suzuki-Miyaura coupling partners and model compounds. Figure S1. IR spectra of (A) 4-iodobenzoic acid (B) model compound (**1**) and (C) boron model compound. Figure S2. IR spectra of (A) 3-(4-bromophenyl)propionic acid (B) model compound (**2**) and (C) boron model compound. Figure S3. IR spectra of (A) model compound (**2**), (B) BNP after reaction with 3-(4-bromophenyl)propionic and (C) BNP. Figure S4. IR spectra of (A) 4-iodo benzaldehyde (B) model compound (**3**) and (C) boron model compound. Figure S5. IR spectra of (A) model compound (**3**), (B) BNP after reaction with 4-iodobenzaldehyde and (C) BNP. Figure S6. IR spectra of (A) Tert-butyl 2-methylcarbazate, (B) Suzuki-Miyaura coupling partner (**4**) (C) model compound (**4**) and (D) boron model compound. Figure S7. IR spectra of (A) model compound (**4**), (B) BNP after reaction with Suzuki-Miyaura-coupling partner (**4**) and (C) BNP. Figure S8. IR spectra of (A) Tert-butyl 2-methylcarbazate, (B) Suzuki-Miyaura coupling partner (**5**) (C) model compound (**5**) and (D) boron model compound. Figure S9. IR spectra of (A) model compound (**5**), (B) BNP after reaction with Suzuki-Miyaura coupling partner (**5**) and (C) BNP. Figure S10. ^1^H NMR spectrum of the boron model compound. Figure S11. ^1^H NMR spectrum of Suzuki-Miyaura coupling partner (**4**). Figure S12. ^1^H NMR spectrum of Suzuki-Miyaura coupling partner (**5**). Figure S13. ^1^H NMR spectrum of model compound (**1**). Figure S14. ^1^H NMR spectrum of model compound (**2**). Figure S15. ^1^H NMR spectrum of model compound (**3**). Figure S16. ^1^H NMR spectrum of model compound (**4**). Figure S17. ^1^H NMR spectrum of model compound (**5**).

## Sample Availability

Samples of the compounds are not available from the authors.
