# Supplementary material for: Functionalization of Framboidal Phenylboronic Acid-Containing Nanoparticles via Aqueous Suzuki–Miyaura Coupling Reactions
Source: Molecules. 2023 Apr 20;28(8):3602. doi: 10.3390/molecules28083602 (PMC10141150; doi:10.3390/molecules28083602)
Supplement: Supplementary file 1 [file molecules-28-03602-s001.zip › molecules-2353091-SI.pdf]

Functionalization of Framboidal Phenylboronic Acid-Containing Nanoparticles via Aqueous Suzuki–Miyaura Coupling Reactions

André J. van der Vlies and Urara Hasegawa *

Department of Materials Science and Engineering, Pennsylvania State University,
331 Steidle Building, University Park, State College, PA 16801, USA; amv5829@psu.edu
* Correspondence: uph5002@psu.edu

SUPPORTING INFORMATION

Page	Figure	Description
1-2	-	Table of content supporting information
3	-	Materials used for synthesis
	-	Synthesis of Suzuki-Miyaura coupling partner (**4**)
4	-	Synthesis of Suzuki-Miyaura coupling partner (**5**)
	-	Synthesis of the boron model compound
5	-	Synthesis of model compound (**1**)
	-	Synthesis of model compound (**2**)
6	-	Synthesis of model compound (**3**)
	-	Synthesis of model compound (**4**)
7	-	Synthesis of model compound (**5**)
8	S1	Infrared spectrum of model compound (**1**)
	S2	Infrared spectrum of model compound (**2**)
9	S3	Infrared spectrum of BNP reacted with Suzuki-Miyaura coupling partner (**2**)
	S4	Infrared spectrum of model compound (**3**)
10	S5	Infrared spectrum of BNP reacted with Suzuki-Miyaura coupling partner (**3**)
	S6	Infrared spectrum of Suzuki-Miyaura coupling partner (**4**)
11	S7	Infrared spectrum of BNP reacted with Suzuki-Miyaura coupling partner (**4**)
	S8	Infrared spectrum of Suzuki-Miyaura coupling partner (**5**)
12	S9	Infrared spectrum of BNP reacted with Suzuki-Miyaura coupling partner (**5**)
	S10	^{1}H NMR spectrum of the boron model compound

13	S11	^{1}H NMR spectrum of Suzuki-Miyaura coupling partner (**4**)
	S12	^{1}H NMR spectrum of Suzuki-Miyaura coupling partner (**5**)
14	S13	^{1}H NMR spectrum of model compound (**1**)
	S14	^{1}H NMR spectrum of model compound (**2**)
15	S15	^{1}H NMR spectrum of model compound (**3**)
	S16	^{1}H NMR spectrum of model compound (**4**)
16	S17	^{1}H NMR spectrum of model compound (**5**)
	References	

Materials

Chemistry. 4-dimethylaminopyridine (DMAP), dicyclohexylcarbodiimide (DCC), 4-morpholineethanesulfonic acid hydrate (MES), ammonium persulfate $(NH_4)_2S_2O_8$ (APS), sodium tetrachloropalladate (Na_2PdCl_4), potassium carbonate (K_2CO_3), potassium hydroxide (KOH), sodium hydroxide (NaOH), propionic acid, triethyl amine (Et_3N), methylenebisacrylamide (MBA), and silica were purchased from Sigma Aldrich. Dichloromethane (CH_2Cl_2), tert-butylalcohol (tert-BuOH), diethyl ether (Et_2O), 1-ethyl-3-(3-dimethylaminopropyl)carbodiimide hydrochloride (EDC), were purchased from Fisher Scientific. Tert-butyl 2-methylcarbazate, sodium diphenylphosphinobenzene-3-sulfonate (PPh_2PhSO_3Na), 4-iodobenzoic acid, 4-bromobenzoic acid, 4-iodobenzaldehyde, 3-(4-bromophenyl)propionic acid, Alizarin Red, were purchased from TCI. Phosphate buffers with pH7 and pH7.4 were from Nacalai Tesque. Deuterated DMSO (d_6-DMSO) and chloroform ($CDCl_3$) were purchased from Cambridge isotopes. Sodium dodecatungstato(VI) phosphate n-hydrate was purchased from Wako. All chemicals and reagents were used as received unless stated otherwise.

APS was recrystallized by cooling a solution in H_2O/EtOH at 4°C. Crystals were collected on a filter and dried under vacuum at room temperature and stored at -20°C until use. MBA was crystallized from MeOH at 4°C. Crystals were vacuum dried and stored at -20°C until use. Triethylamine was distilled from ninhydrin and stored in the dark over KOH pellets. Sodium dodecatungstato(VI) phosphate solution (2 wt%) was prepared by dissolving sodium dodecatungstato(VI) phosphate n-hydrate in water and adjusting the pH to 7-8 with 1 M NaOH. (5-(4-(2-aminoethoxy)phenyl)-3H-1,2-dithiole-3-thione) TFA salt ($ADT\text{-}NH_2 \cdot TFA$)[1], PEG-acrylamide (PEG-AM = 5Da)[2] and the PAPBA monomer[2] were prepared as reported.

Synthesis of Suzuki-Miyaura coupling partner (4)

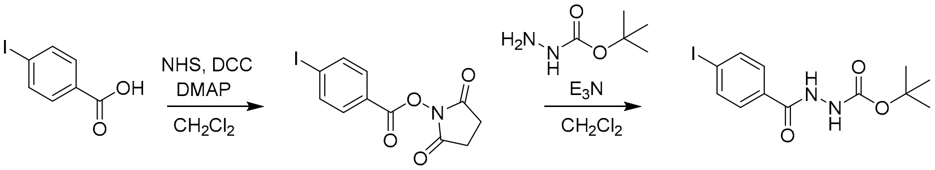

4-iodobenzoic acid (496 mg, 2.0 mmol, 1 eq), NHS (244 mg, 2.1 mmol, 1.05 eq) and DMAP (12.9 mg, 0.11 mmol, 0.05 eq) were suspended in 50 mL dry CH_2Cl_2 and cooled at 0°C for 30 min. DCC (433 mg, 2.1 mmol, 1.05 eq) was added in 2 mL dry CH_2Cl_2 and after 1 h the mixture was stirred at room temperature. After stirring for another 4 h, TLC indicated the formation of the NHS ester and tert-butyl 2-methylcarbazate (278 mg, 2.1 mmol, 1.05 eq) was added followed by Et_3N (293 µL, 2.1 mmol, 1.05 eq). After 17 h the mixture was filtered over a plug of glass wool and the clear filtrate concentrated under reduced pressure. The residue was suspended in 50 mL EtOAc, filtered, and washed with 5% $NaHCO_3$ (aq) (3 x 50 mL), 1 M $NaHSO_4$ (aq) (3 x 50 mL) and water (50 mL). After drying over Na_2SO_4 the solution was

concentrated under reduced pressure. The residue was suspended in 14 mL CH_2Cl_2, filtered and washed with CH_2Cl_2 (2 x 2 mL) to remove some dicyclohexylurea, After solvent removal the residue was purified by column chromatography (EtOAc/hexane 1:2) to yield 419 mg (1.2 mmol, 58%) of Suzuki-Miyaura coupling partner (**4**) as a white solid that is soluble in polar solvents like methanol and dimethylsulfoxide.
^{1}H NMR (d_6-DMSO): δ in ppm = 10.26 (s, 1H, NH), 8.94 (bs, 1H, NH), 7.88 (d, 2H, 2 x CH_{aromat}), 7.62 (d, 2H, 2 x CH_{aromat}),1.42 (s, 9H, $C(CH_3)_3$).

Synthesis of Suzuki-Miyaura coupling partner (5)

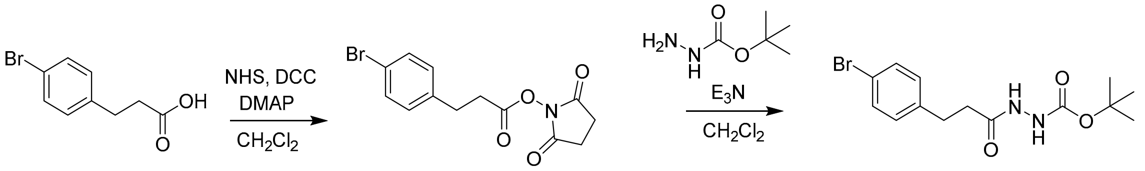

3-(4-bromophenyl)propionic acid (458 mg, 2.0 mmol), NHS (245 mg, 2.1 mmol, 1.05 eq) and DMAP (12.2 mg, 0.1 mmol, 0.05 eq) were dissolved in dry CH_2Cl_2 and cooled at 0°C for 1 h before adding DCC (439.0 mg, 2.1 mmol, 1.05 eq) in 2 mL dry CH_2Cl_2. After 7 h TLC of the white suspension showed formation of the NHS ester, and tert-butyl 2-methylcarbazate (280 mg, 2.1 mmol, 1.05 eq) was added followed by Et_3N (293 µL, 2.1 mmol, 1.05 eq). After stirring the mixture for 20 h the suspension was filtered, and the white residue washed with CH_2Cl_2 (2 x 2 mL). After CH_2Cl_2 removal the residue was suspended in 60 mL EtOAc, filtered, and washed with 5% $NaHCO_3$ (aq) (3 x 50 mL), 1 M $NaHSO_4$ (aq) (3 x 50 mL), dried over Na_2SO_4 and concentrated under reduced pressure. The residue was purified by column chromatography (EtOAc/hexane 2:3) to yield 522 mg (1.5 mmol, 76%) of Suzuki-Miyaura coupling partner (**5**) as a white solid that is soluble in polar solvents like methanol and dimethylsulfoxide.
^{1}H NMR (d_6-DMSO) δ in ppm = 9.54 (s, 1H, NH), 8.70 (s, 1H,), 7.44 (d, 2H, 2 x CH_{aromat}), 7.18 (d, 2H, 2 x CH_{aromat}), 2.79 (m, 2H, CH_2), 2.36 (m, 2H, CH_2), 1.39 (s, 9H, $C(CH_3)_3$).

Synthesis of boron model compound

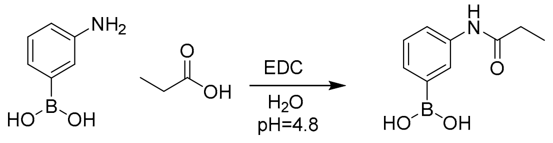

(3)

3-aminophenylboronic acid monohydrate (1.2 g, 7.9 mmol) and propionic acid (600, µL, 8.0 mmol) were dissolved in 20 mL water. After adjusting the pH to 4.8 with 0.1 M NaOH (aq) the solution was cooled in ice water before adding EDC (1.6 g, 8.2 mmol). After 2 h the reaction mixture was extracted with Et_2O (3 x 50 mL), dried over Na_2SO_4 and concentrated under reduced pressure. The residue was suspended in 50

mL water and dissolved by heating. The clear solution was separated from some sticky insoluble material by decantation and kept at 4ºC for 2 days. The crystals were filtered and dried under vacuum to yield 645 mg (3.3 mmol, 42%) of boron model compound as a yellow solid that is soluble in polar solvents like methanol and dimethylsulfoxide. ^{1}H NMR (d_6-DMSO): δ in ppm = 9.78 (s, 1H, NH), 8.00 (s, 2H, $B(OH)_2$), 7.82 (s, 1H, CH_{aromat}), 7.71 (d. 1H, CH_{aromat}), 7.44 (m, 1H, CH_{aromat}), 7.25 (m, 1H, CH_{aromat}), 2.29 (q, 2H, CH_2), 1.07 (t, 3H, CH_3).

Synthesis of model compound (1)

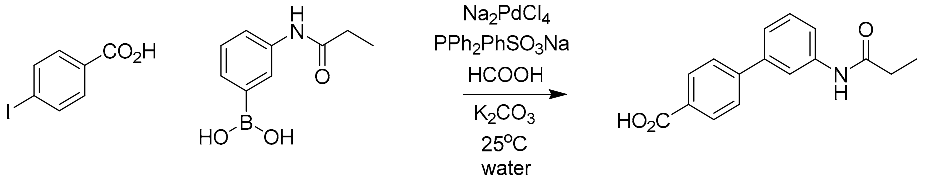

4-iodobenzoic acid (61 mg, 0.25 mmol, 1 eq), boron model compound (51 mg, 0.26 mmol, 1.1 eq) and K_2CO_3 (71 mg, 0.52 mmol, 2.1 eq) were dissolved in 10 mL degassed water. After evacuating and purging with argon an aqueous solution (100 µL) containing Na_2PdCl_4 (0.01 eq) and PPh_2PhSO_3Na (molar ratio phosphine/Pd = 41) was added followed by 100 µL of an aqueous solution of HCOOH (0.05 eq). The mixture was stirred at the room temperature and workup as described above. The reaction mixture was acidified with 1 M $NaHSO_4$ (aq) (1 mL) and the suspension filtered on a glass filter. After washing with H_2O (3 x 30 mL) the solid was dissolved in acetone (30 mL) and after solvent removal dried under vacuum. This yielded 65 mg (0.24 mmol, 96%) of model compound (**1**) as a white solid that is soluble in polar solvents like methanol and dimethylsulfoxide.

^{1}H NMR (d_6-DMSO): δ in ppm = 13.0 (s, 1H, COOH), 10.0 (s, 1H, NH), 8.04-8.02 (m, 3H, 3 x CH_{aromat}), 7.74-7.62 (m, 2H, 2 x CH_{aromat}), 7.61 (s, 1H, CH_{aromat}), 7.42-7.39 (m, 2H, 2 x CH_{aromat}), 2.34 (q, 2H, CH_2), 1.10 (t, 3H, CH_3).

Synthesis of model compound (2)

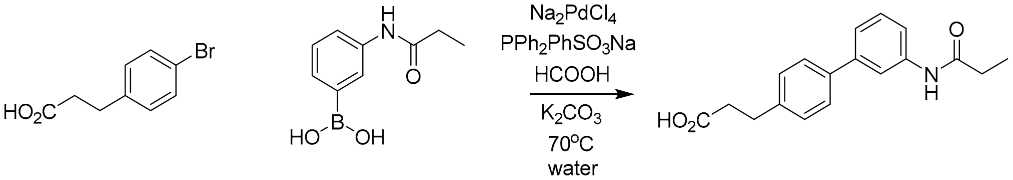

3-(4-bromophenyl)propionic acid (56 mg, 0.25 mmol, 1.0 eq), boron model compound (51 mg, 0.26 mmol, 1.05 eq) and K_2CO_3 (73 mg, 0.53 mmol, 2.1 eq) were dissolved in 10 mL degassed water. After evacuating and purging with argon an aqueous solution (100 µL) containing Na_2PdCl_4 (0.01 eq) and PPh_2PhSO_3Na (molar ratio phosphine/Pd = 4) was added followed by 100 µL of an aqueous solution of HCOOH (0.05

eq). After stirring at 70°C for 19 h, the reaction mixture was diluted with 20 mL 1 M $NaHSO_4$ (aq). The beige suspension was filtered, and the residue washed with water (3 x 30 mL). After dissolving in acetone and solvent removal this yielded model compound (**2**) in quantitative yield as a white solid that is soluble in polar solvents like methanol and dimethylsulfoxide.

^1H NMR (d_6-DMSO): δ (in ppm) = 12.16 (bs, 1H, COOH), 9.94 (s, 1H, NH), 7.89 (bs, 1H, CH_{aromat}), 7.55 (d, 1H, CH_{aromat}), 7.51 (d, 2H, 2 x CH_{aromat} para substituted), 7.37 (d, 1H, CH_{aromat}), 7.32 (d, 2H, CH_{aromat} para substituted), 7.28 (d, 1H, CH_{aromat}), 2.86 (t, 2H, CH_2), 2.57 (t, 2H, CH_2), 2.34 (q, 2H, $C\underline{H_2}CH_3$), 1.09 (t, 3H, CH_3).

Synthesis of model compound (3)

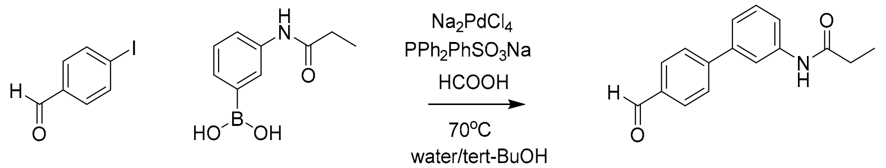

4-Iodobenzaldehyde (53 mg, 0.23 mmol, 1 eq), boron model compound (57 mg, 0.30 mmol, 1.3 eq) and K_2CO_3 (35 mg, 0.25 mmol, 1.1 eq) were dissolved in 4 mL degassed tert-BuOH/water (3/1). After evacuating and purging with argon an aqueous solution (100 µL) containing Na_2PdCl_4 (0.01 eq) and PPh_2PhSO_3Na (molar ratio phosphine/Pd = 4) was added followed by 100 µL of an aqueous solution of HCOOH (0.05 eq). After stirring at 70°C for 24 h the reaction mixture was diluted with 25 mL water. The suspension was filtered, and the solid washed with water (3 x 25 mL). The solid was dissolved in acetone and after solvent removal the residue was purified by silica column chromatography eluting with EtOAc to yield 55 mg (0.22 mmol, 95%) of model compound (**3**) as a white solid that is soluble in polar solvents like methanol and dimethylsulfoxide.

^1H NMR (d_6-DMSO): δ in ppm = 10.06 (s, 1H, aldehyde), 10.02 (s, 1H, NH), 8.04-7,99 (m, 3H, 3 x CH_{aromat}), 7.85-7,83 (m, 2H, 2 x CH_{aromat}), 7.64-7.61 (m, 1H, CH_{aromat}), 7.45-7.42 (m, 2H, 2 x CH_{aromat}), 2.35 (q, 2H, CH_2), 1.10 (t, 3H, CH_3).

Synthesis of model compound (4)

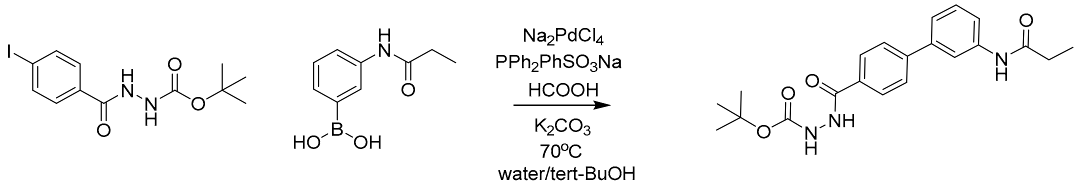

Suzuki-Miyaura coupling partner (**4**) (98 mg, 0.27 mmol, 1 eq) and boron model compound (55 mg, 0.28 mmol, 1.05 eq) and K_2CO_3 (39 mg, 0.28 mmol, 1.05 eq). were dissolved in 0.7 mL water and 3 mL *tert*-

BuOH. After evacuating and purging with argon an aqueous solution (100 µL) containing Na_2PdCl_4 (0.01 eq) and PPh_2PhSO_3Na (molar ratio phosphine/Pd = 4) and 100 µL of an aqueous solution of HCOOH (0.05 eq) were added. After stirring at 70°C for 24 h the reaction mixture was diluted with 25 mL water and filtered. The residue was washed with water (3 x 25 mL), dried and dissolved in acetone. After removing the solvent the residue was purified over a short silica column eluting with EtOAc. After solvent removal the solid (77.5 mg) was dissolved in warm EtOAc and put at 40°C. The white crystals that formed were filtered and dried to yield model compound (**4**) (42 mg, 0.11 mmol, 40%) as a white solid that is soluble in polar solvents like methanol and dimethylsulfoxide.

^{1}H NMR (d_6-DMSO): δ in ppm = 10.24 (s, 1H, $NH_{hydrazide}$), 9.98 (s, 1H, NH_{amide}), 8.93 (s, 1H, $NH_{hydrazide}$), 8.00 (bs, 1H, CH_{aromat}), 7.96 (d, 2H, 2 x CH_{aromat}), 7,72 (d, 2H, 2 x CH_{aromat}), 7.61 (d, 1H, CH_{aromat}), 7.41-738 (m, 2H, 2 x CH_{aromat}), 2.35 (t, 2H, CH_2), 1.44 (bs, 9H, $C(CH_3)_3$), 1.10 (t, 3H, CH_3).

Synthesis of model compound (5)

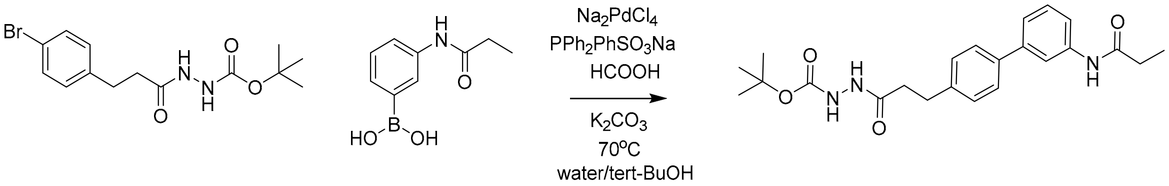

Suzuki-Miyaura coupling partner (**5**) (82.3 mg, 0.24 mmol, 1 eq) and boron model compound (56mg, 0.29 mmol, 1.4 eq) were dissolved in 3 mL tert-BuOH and a solution of K_2CO_3 (41 mg, 0.29 mmol, 1.4 eq) in water (0.8 mL) was added. After evacuating and purging with argon an aqueous solution (100 µL) containing Na_2PdCl_4 (0.01 eq) and PPh_2PhSO_3Na (molar ratio phosphine/Pd = 4) and 100 µL of an aqueous solution of HCOOH (0.05 eq) were added. After stirring at 70°C for 24 h the reaction mixture was diluted with 25 mL water and filtered. The residue was washed with water (3 x 25 mL). After drying the residue was dissolved in acetone and after solvent removal passed over silica eluting with EtOAc. to yield model compound (**5**) in quantitative yield as a white solid that is soluble in polar solvents like methanol and dimethylsulfoxide.

^{1}H NMR (d_6-DMSO): δ in ppm = 9.94 (s, 1H, $NH_{hydrazide}$), 9.58 (s, 1H, NH_{amide}), 8.72 (s, 1H, $NH_{hydrazide}$), 7.89 (bs, 1H, CH_{aromat}), 7.55 (d, 2H, 2 x CH_{aromat}), 7.51 (d, 2H, 2 x CH_{aromat}), 7.37 (d, 1H, CH_{aromat}), 7.32-7.27 (m, 2H, 2 x CH_{aromat}), 2.86 (t, 2H, CH_2), 2.41 (t, 2H, CH_2), 2.34 (t, 2H, CH_2), 1.40 (bs, 9H, $C(CH_3)_3$), 1.09 (t, 3H, CH_3).

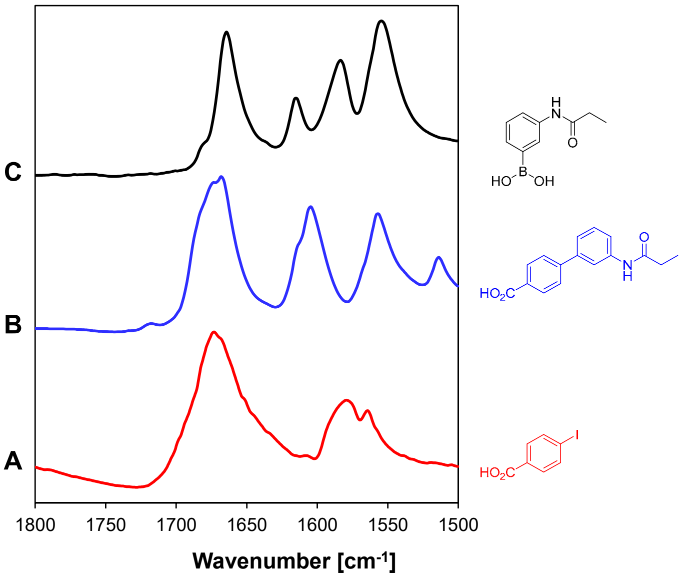

Figures S1. IR spectra of (**A**) 4-iodobenzoic acid (**B**) model compound (**1**) and (**C**) boron model compound.

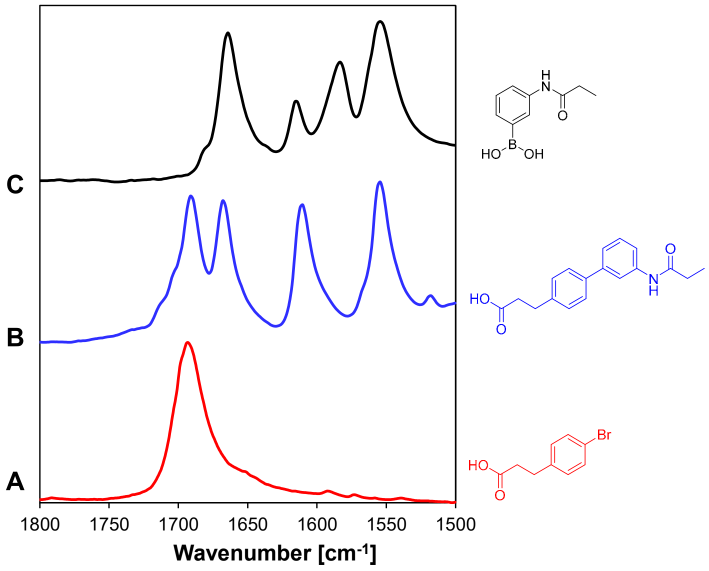

Figure S2. IR spectra of (**A**) 3-(4-bromophenyl)propionic acid (**B**) model compound (**2**) and (**C**) boron model compound.

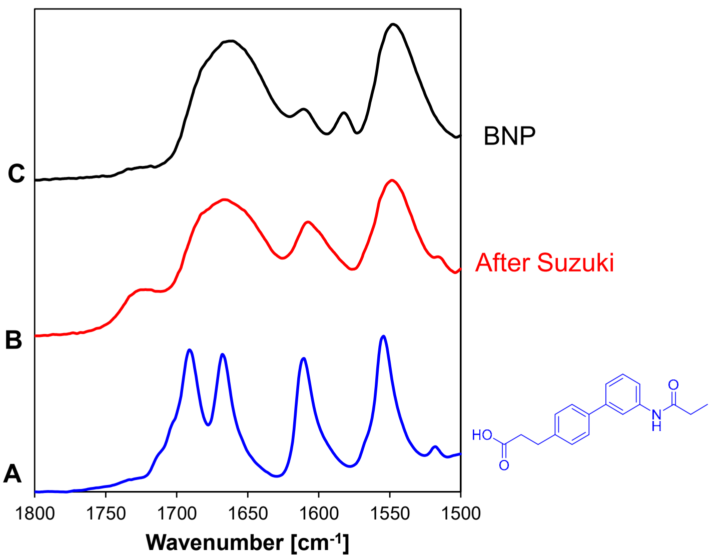

Figure S3. IR spectra of (**A**) model compound (**2**), (**B**) BNP after reaction with 3-(4-bromophenyl)propionic and (**C**) BNP.

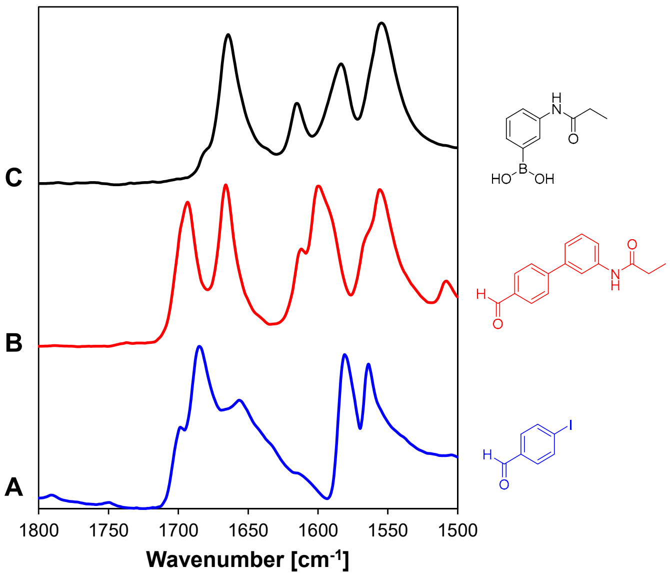

Figure S4. IR spectra of (**A**) 4-iodo benzaldehyde (B) model compound (**3**) and (**C**) boron model compound.

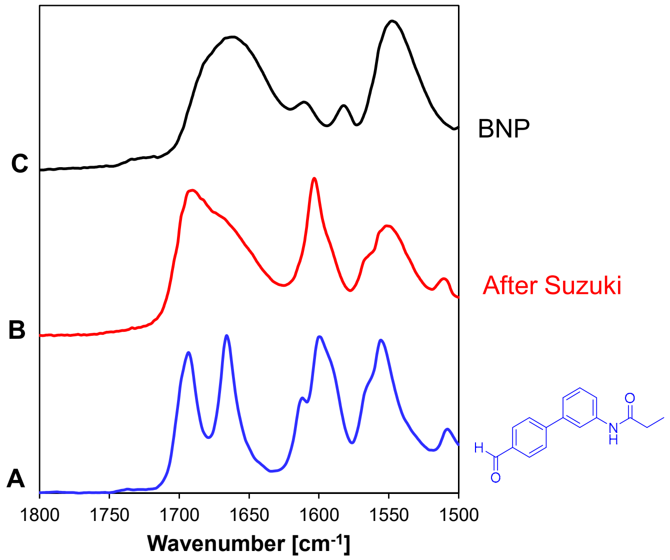

Figure S5. IR spectra of (**A**) model compound (**3**), (**B**) BNP after reaction with 4-iodobenzaldehyde and (**C**) BNP.

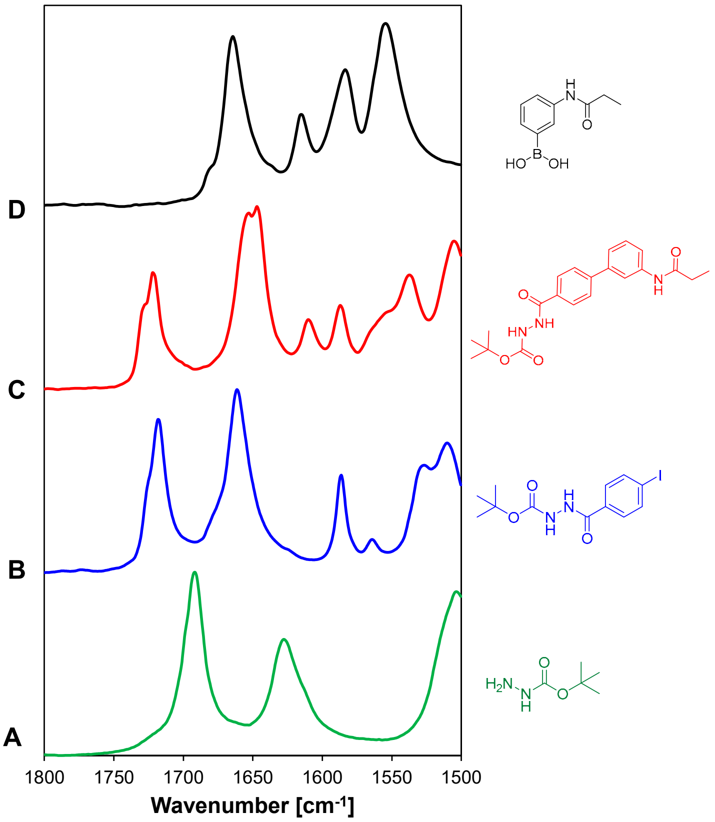

Figure S6. IR spectra of (**A**) Tert-butyl 2-methylcarbazate, (**B**) Suzuki-Miyaura coupling partner (**4**) (**C**) model compound (**4**) and (**D**) boron model compound.

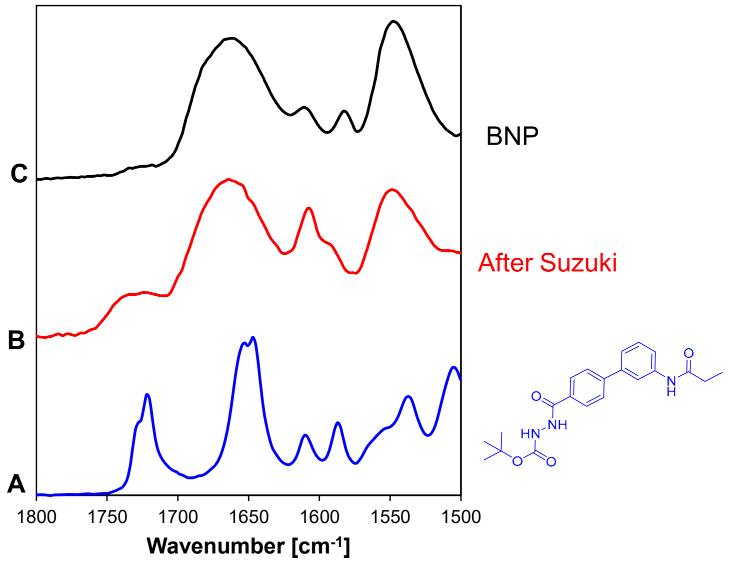

Figure S7. IR spectra of (**A**) model compound (**4**), (**B**) BNP after reaction with Suzuki-Miyaura-coupling partner (**4**) and (**C**) BNP.

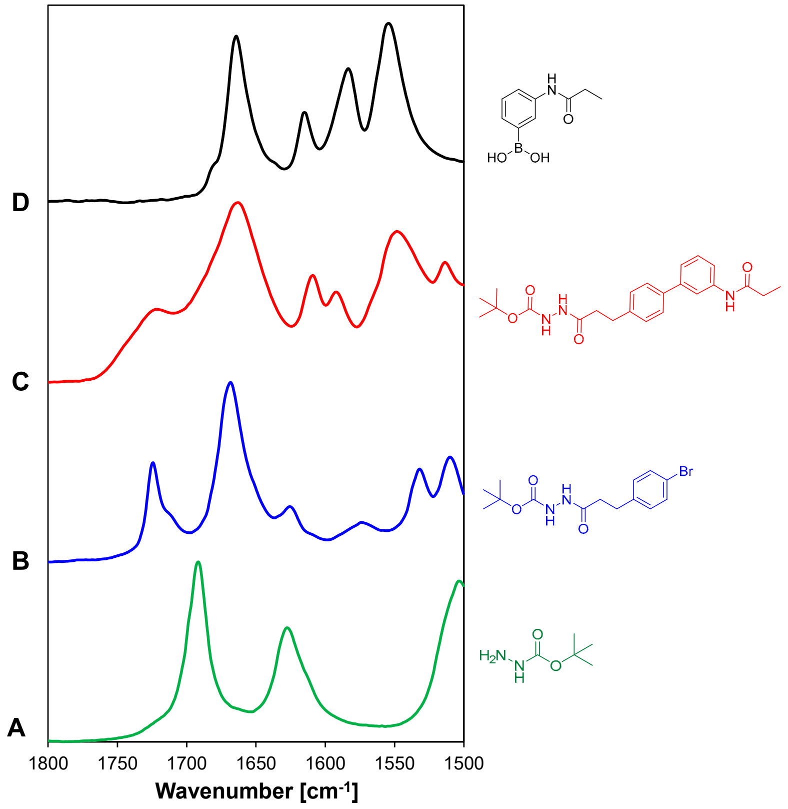

Figure S8. IR spectra of (**A**) Tert-butyl 2-methylcarbazate, (**B**) Suzuki-Miyaura coupling partner (**5**) (**C**) model compound (**5**) and (**D**) boron model compound.

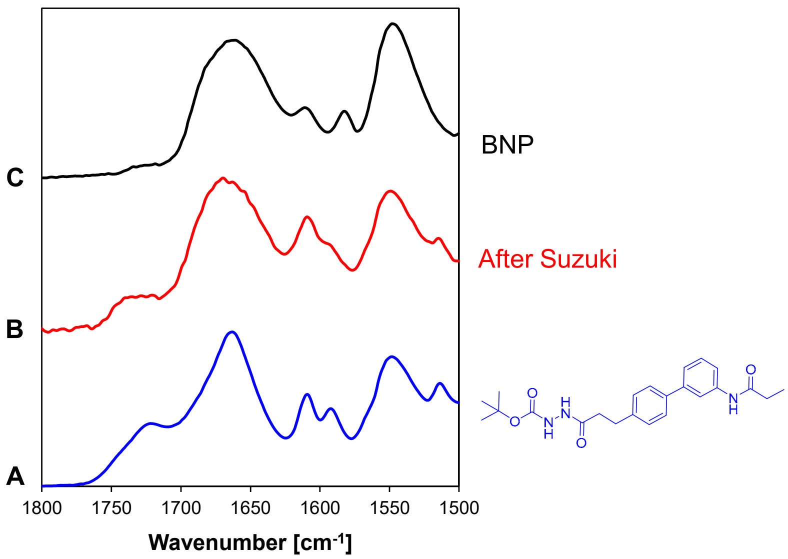

Figure S9. IR spectra of (**A**) model compound (**5**), (**B**) BNP after reaction with Suzuki-Miyaura coupling partner (**5**) and (**C**) BNP.

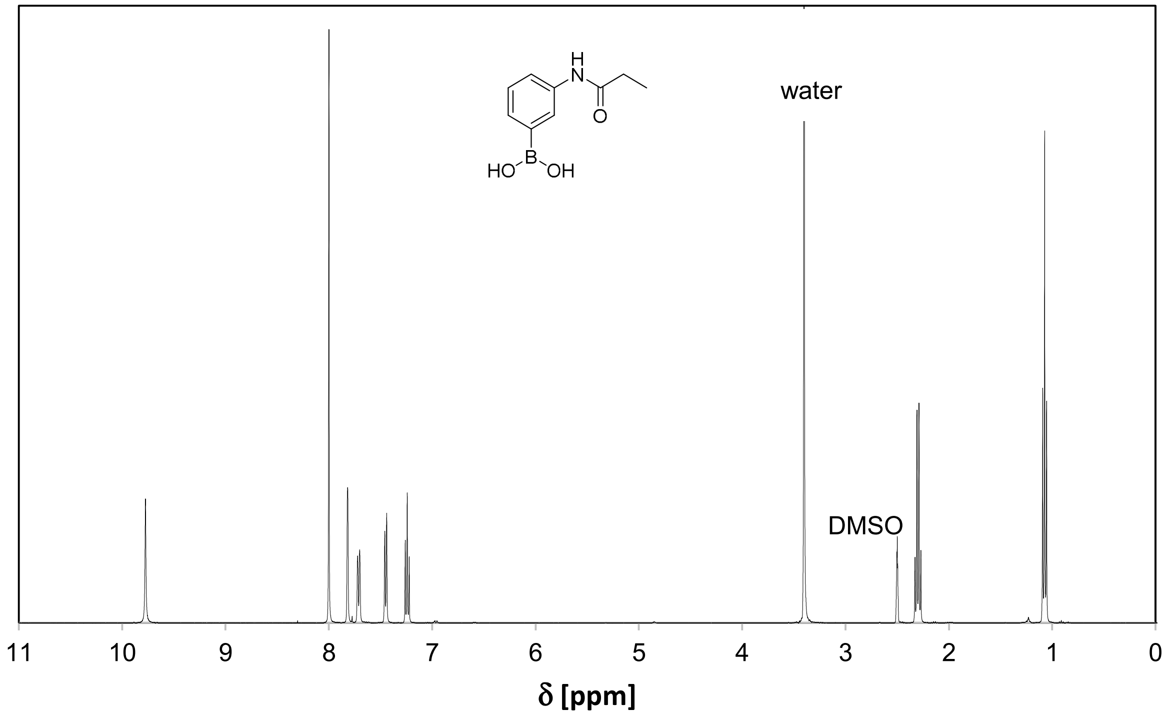

Figure S10. ^{1}H NMR spectrum of the boron model compound.

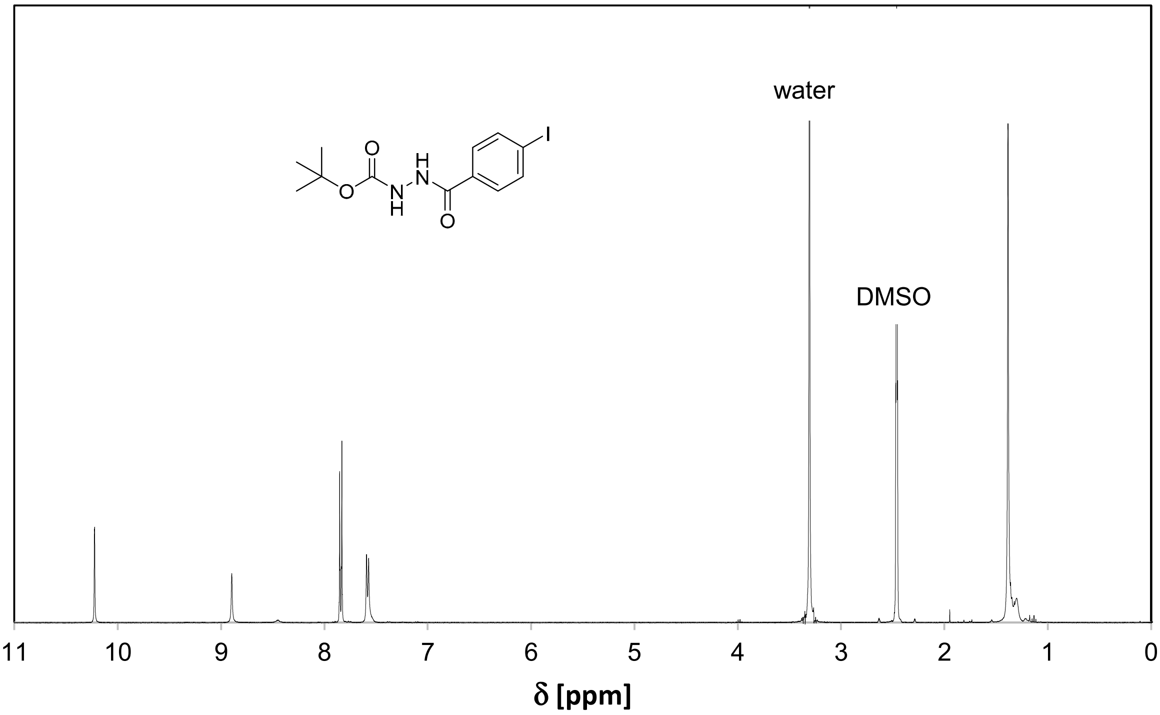

Figure S11. ^{1}H NMR spectrum of Suzuki-Miyaura coupling partner (**4**)

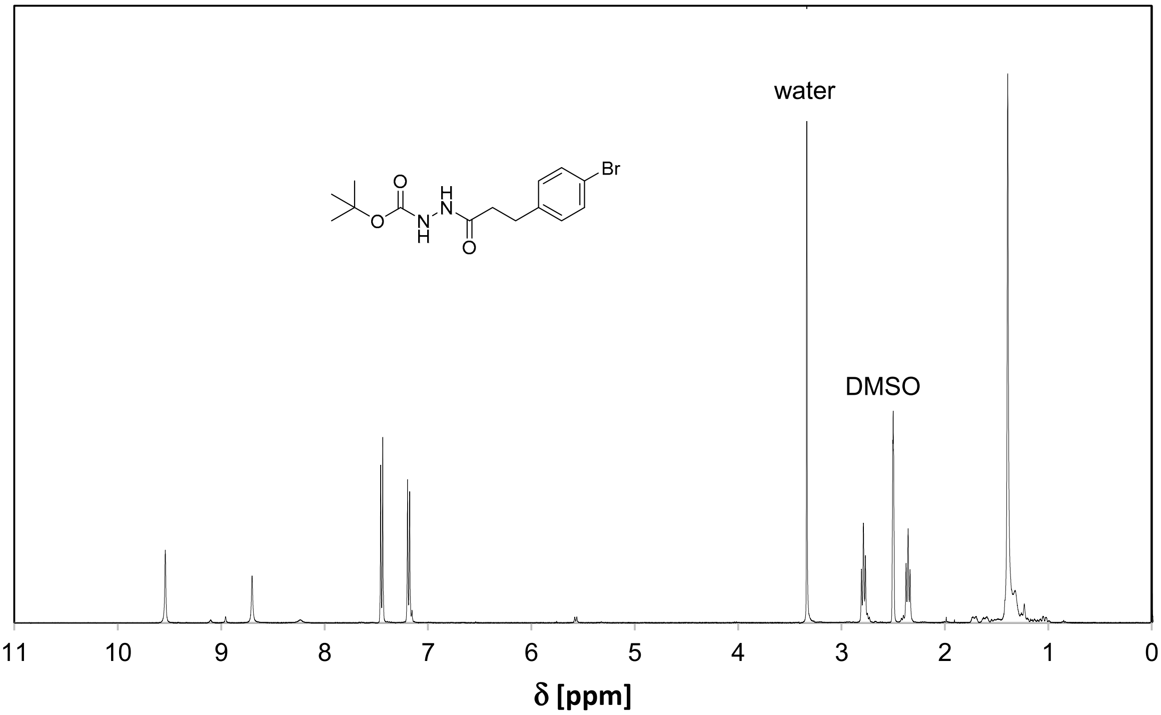

Figure S12. ^{1}H NMR spectrum of Suzuki-Miyaura coupling partner (**5**)

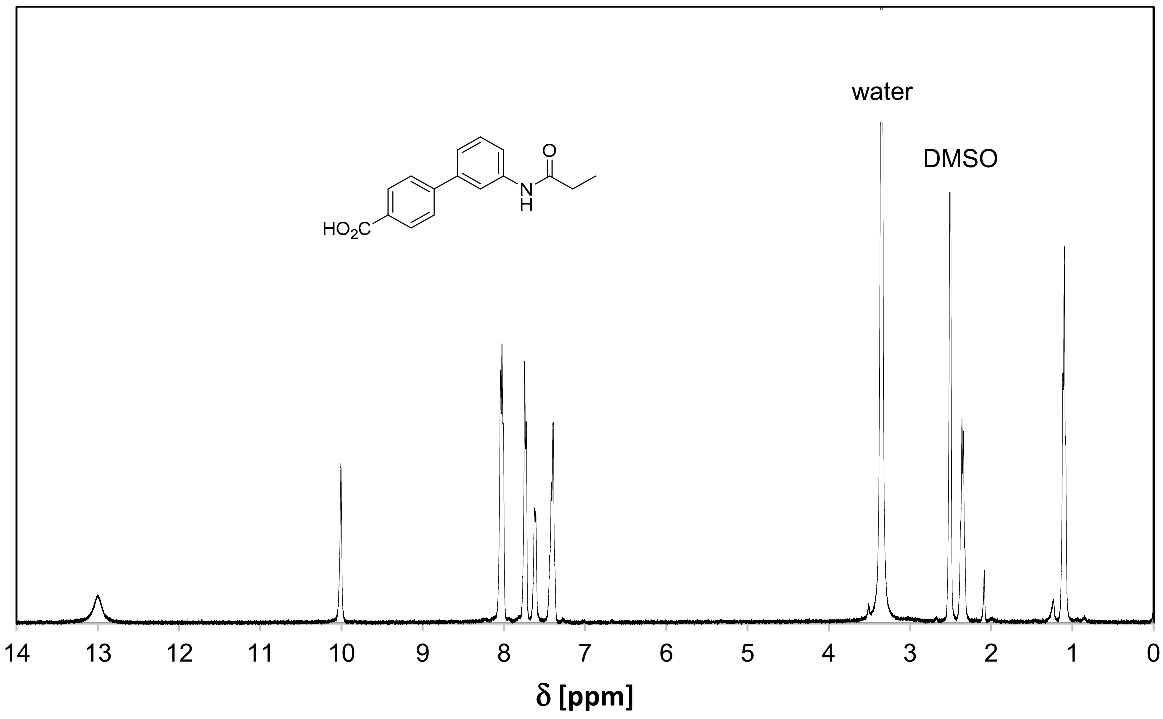

Figure S13. ^{1}H NMR spectrum of model compound (**1**)

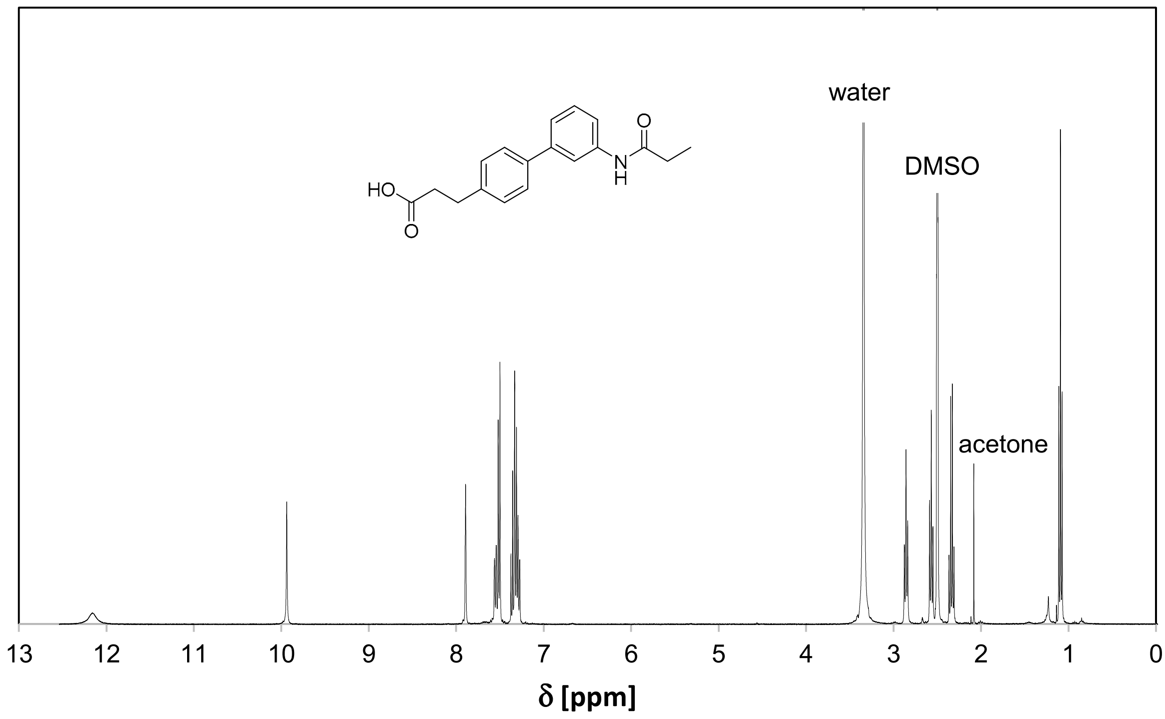

Figure S14. ^{1}H NMR spectrum of model compound (**2**)

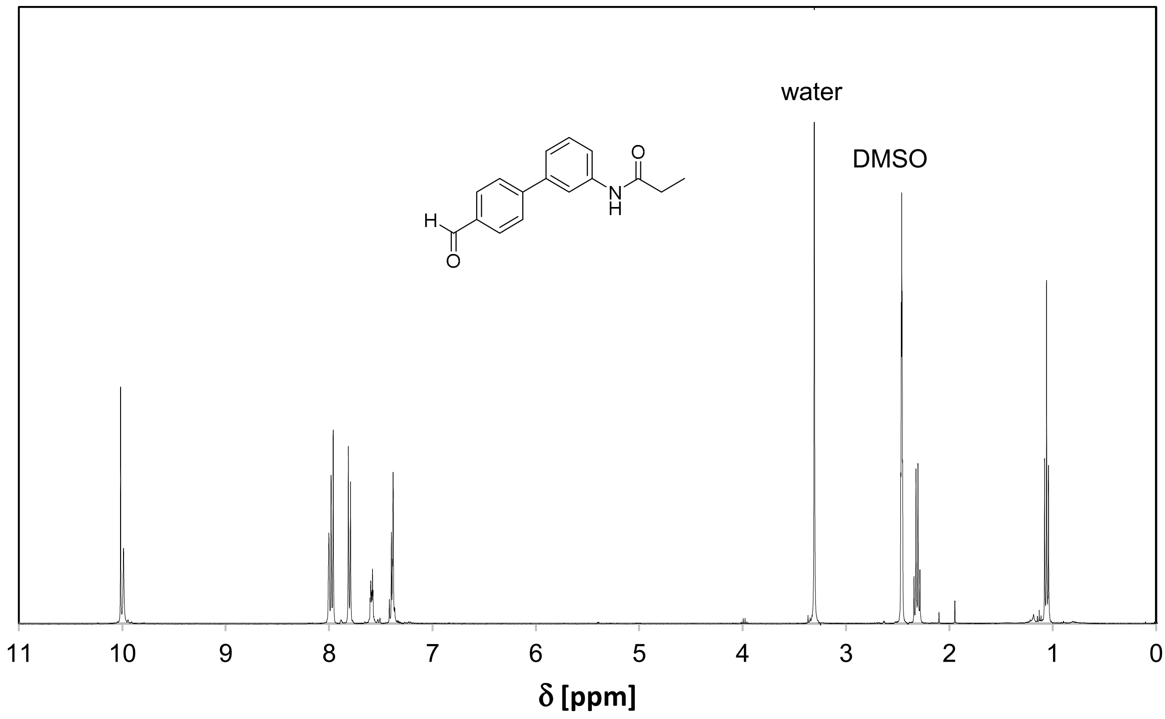

Figure S15. ^{1}H NMR spectrum of model compound (**3**)

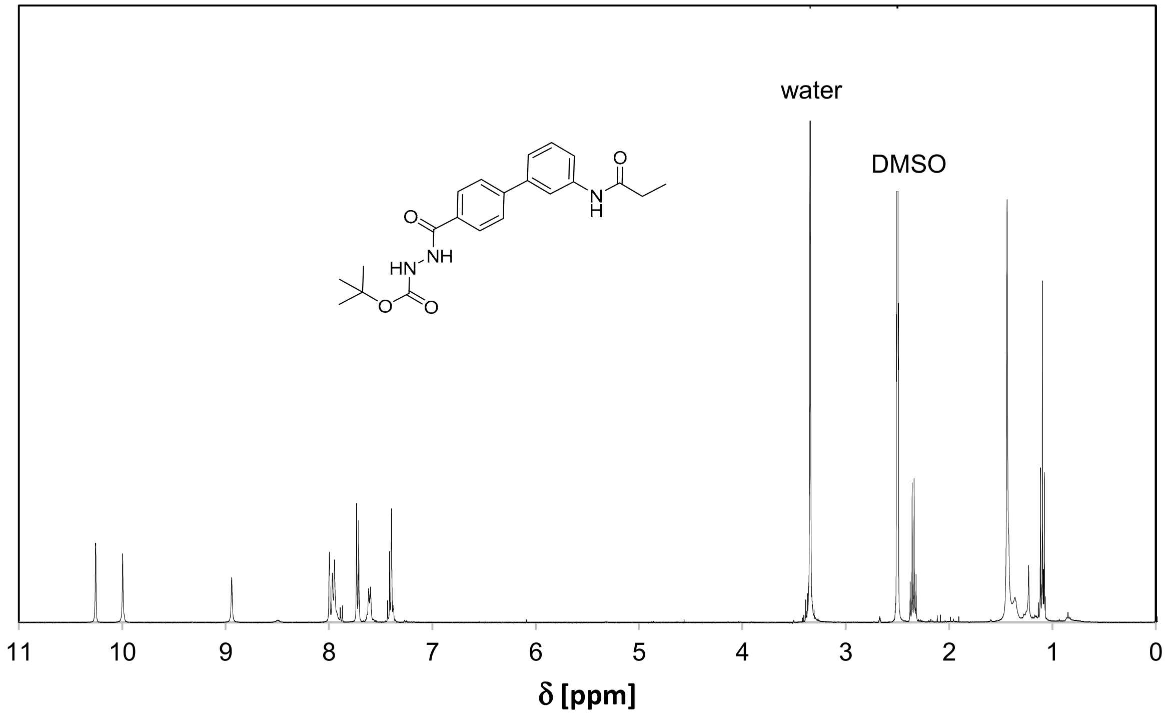

Figure S16. ^{1}H NMR spectrum of model compound (**4**)

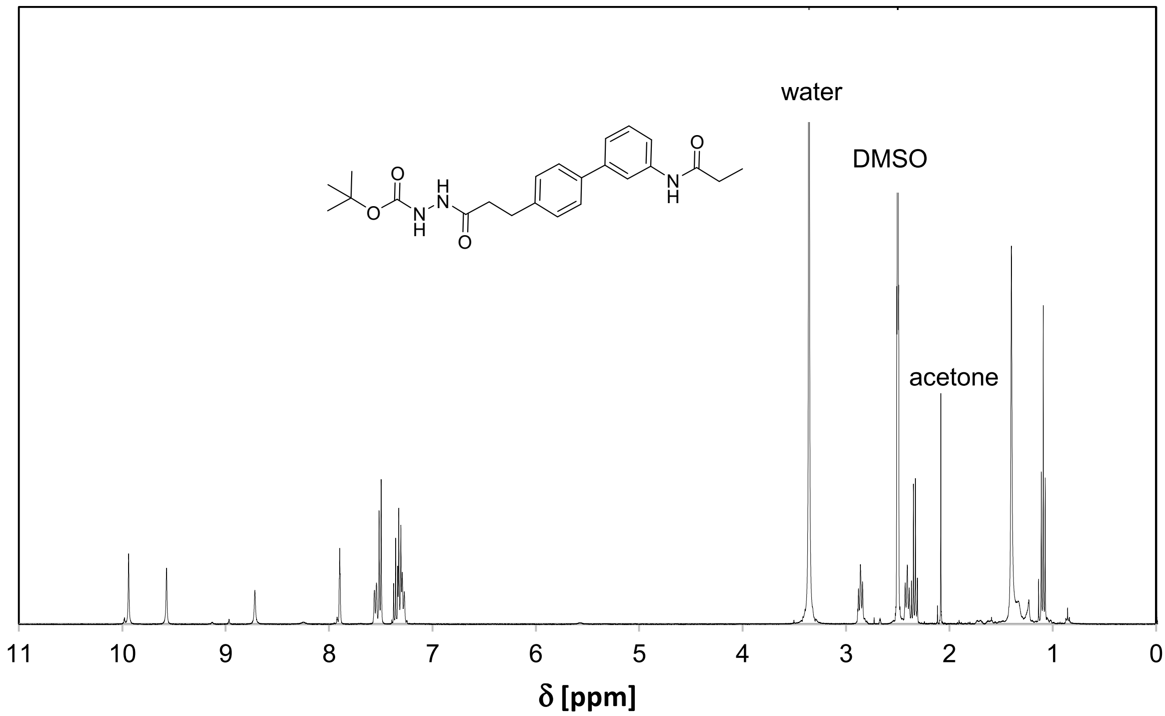

Figure S17. ^{1}H NMR spectrum of model compound (**5**)

References

1. Hasegawa, U.; van der Vlies, A. J., Polymeric micelles for hydrogen sulfide delivery. *MedChemComm* **2015,** 6, (2), 273-276.
2. Hasegawa, U.; Inubushi, R.; Uyama, H.; Uematsu, T.; Kuwabata, S.; van der Vlies, A. J., Mannose-displaying fluorescent framboidal nanoparticles containing phenylboronic acid groups as a potential drug carrier for macrophage targeting. *Colloids and Surfaces B: Biointerfaces* **2015,** 136, 1174-1181.
